# Endomyocardial fibrosis in a non‐tropical patient who presented with chest pain mimicking ACS and left ventricular thrombus, case report

**DOI:** 10.1002/ccr3.5021

**Published:** 2022-05-23

**Authors:** Ahmad S. Matarneh, Yousef M. Ali Hailan, Sabir Abdul Karim, Maryam A. Al Kuwari, Wafer A. Dabdoob

**Affiliations:** ^1^ Internal Medicine Department of Medical Education Hamad Medical Corporation (HMC) Doha Qatar; ^2^ Department of Cardiology Heart Hospital HMC Doha Qatar; ^3^ Heart Hospital HMC Doha Qatar

**Keywords:** cardiovascular diseases, endomyocardial fibrosis, treatment, tropical disease

## Abstract

Endomyocardial fibrosis (EMF) is a disease known to cause restrictive cardiomyopathy. It shows a high prevalence in tropical countries. Several triggering factors have been proposed. However, the pathogenesis is still a mystery. The disease is progressive, and the outcome is generally unfavorable. The most common symptom is heart failure. However, an atypical presentation may be expected. Our case presented with symptoms suggestive of ischemia and missed diagnosis initially as ischemic cardiomyopathy. This report aims to increase the attention and awareness of this disease. We present a case of a 53‐year‐old man referred to the emergency department for sudden chest pain, left‐sided and non‐radiating lasted for several minutes, awoke him from sleep with no associated symptoms. He is known to have Diabetes type‐2 and hypertension on oral therapy. Cardiac markers were within the normal limit. The patient was discharged home with an appointment at the cardiology outpatient clinic. Echocardiography was done and revealed mildly reduced left ventricular (LV) systolic function, Ejection Fraction of 46%, asymmetric LV hypertrophy affecting the apical segments with aneurysm, and calcified apical thrombus. CT coronary angiography was done with non‐significant Left Anterior Descending artery lesions and left ventricular hypertrophy affecting the apex with calcified apical thrombus. Further investigation by cardiac MRI revealed apical thrombus and late apical uptake suggesting Endomyocardial Fibrosis of possible eosinophilic etiology. The patient continued to have attacks of similar chest pain, for which stress cardiac MRI was done and was negative for ischemia. Another diagnostic workup was done, including hematological and serological tests such as Antinuclear Antibodies and Schistosoma Antibodies. The patient was kept on valsartan and Bisoprolol with oral anticoagulant (vitamin K antagonist) and Rosuvastatin. EMF may have a heterogeneous presentation and should be considered in a patient with calcific apical thrombus without previous history of cardiac problem, even in the non‐tropical region.

## Graphical abstract

Endomyocardial fibrosis is a rare cause of restrictive cardiomyopathy. Its etiology is poorly understood with a challenging diagnosis and treatment. It should be considered as a cause of unexplained chest pain and heart failure.

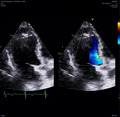

## 1 INTRODUCTION

Endomyocardial fibrosis (EMF) is progressive restrictive cardiomyopathy that usually affects the left, right, or both ventricles. It can also affect the outflow of the ventricles, leading to the development of symptoms of failure of the involved ventricle.^1^ The restrictive involvement is usually seen because of apical fibrosis, which generally arises from collagen deposition and fibroblast proliferation. It is prevalent in tropical and subtropical parts of the world. The leading cause of it is still unknown. However, there are multiple theories available regarding its development.^2^ Treatment is challenging as currently there are limited data; medical therapy as a case of restrictive heart failure might offer symptomatic relief. However, the disease is usually progressive, and the long‐term outcome is generally poor. On the other hand, surgical resection of the endocardium might offer a definite treatment. However, there is still a risk of recurrence, and it carries a high mortality rate.^3^

We report a patient who presented with chest pain and, on subsequent investigations, was diagnosed with EMF.

## 2 CASE PRESENTATION

**FIGURE 1:**
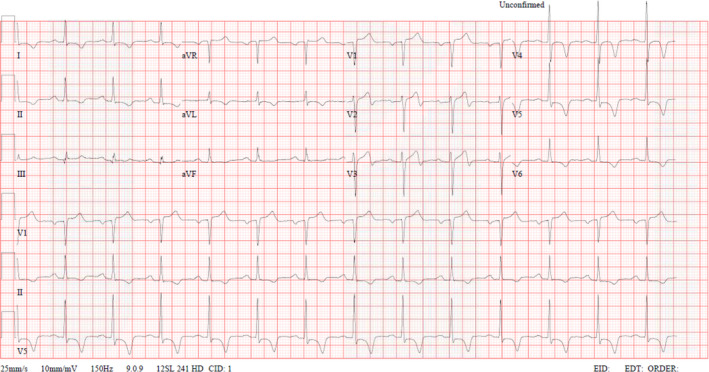
ECG on presentation

A 53‐year‐old male patient from the Eastern Mediterranean Region with a background of diabetes mellitus type II (DM II) and hypertension (HTN), both controlled with oral medications, presented to the primary healthcare center because of acute onset chest pain. Chest pain lasted for several minutes and was left‐sided and non‐radiating. There were no associated symptoms and no specific worsening or relieving factors. Family history is non‐significant. On examination, the patient was afebrile, had a normal pulse and respiratory rates (HR 78, RR 16), blood pressure was high at 155/94 mmHg. Systemic examination was unremarkable. ECG (Figure 1) showed sinus rhythm with T‐inversion in I, II, avl, V3–V6, which were considered old compared to the ECG in 2017. The patient was transferred rapidly to the emergency department (ED) for further evaluation and management through the emergency ambulance service.

The patient was kept under observation; he was chest pain‐free with stable vital signs and physical examination. Chest radiographs and laboratory investigations were unremarkable, including two sets of high sensitivity cardiac troponin T (Troponin T HS) with 7 ng/L and 8 ng/L, respectively. The rest of the laboratory panel areas are mentioned in Table 1. The patient was discharged with cardiology outpatient follow‐up advice.

**TABLE 1:** Laboratory data

Detail	Value w/units	Normal range
WBC	8.0 × 10^3^/μL	4.0–10.0
RBC	5.0 × 10^6^/μL	4.5–5.5
Hgb	15.4 gm/dL	13.0–17.0
Hematocrit	44.2%	40.0–50.0
MCV	88.8 fL	83.0–101.0
MCH	30.9 pg	27.0–32.0
MCHC	34.8 gm/dL	31.5–34.5
RDW‐CV	11.9%	11.6–14.5
Platelet	168 × 10^3^/μL	150–400
MPV	9.7 fL	7.4–10.4
Absolute neutrophil count auto# (ANC)	4.6 × 10^3^/μL	2.0–7.0
Lymphocyte auto #	2.2 × 10^3^/μL	1.0–3.0
Monocyte auto #	0.5 × 10^3^/μL	0.2–1.0
Eosinophil auto #	0.4 × 10^3^/μL	0.0–0.5
Basophil auto #	0.08 × 10^3^/μL	0.02–0.10
Neutrophil auto %	58.4%	
Lymphocyte auto %	28.2%	
Monocyte auto %	6.7%	
Eosinophil auto %	5.7%	
Basophil auto %	1.0%	
Prothrombin time	11.2 s	9.7–11.8
INR	1.0	
APTT	27.3 s	24.6–31.2
Urea	5.7 mmol/L	2.8–8.1
Creatinine	66 umol/L	62–106
Sodium	137 mmol/L	136–145
Potassium	4.2 mmol/L	3.5–5.1
Chloride	101 mmol/L	98–107
Bicarbonate	25 mmol/L	22–29
Calcium	2.23 mmol/L	2.15–2.50
Calcium corrected	2.31 mmol/L	2.15–2.50
Bilirubin, total	14 μmol/L	0–21
Total protein	66 gm/L	66–87
Albumin level	36 gm/L	35–52
Alkaline phosphatase	85 U/L	40–129
ALT	16 U/L	0–41
AST	12 U/L	0–40
NT pro‐BNP	85 pg/mL	
Troponin T HS	7 ng/L	3–15
Cholesterol	3.7 mmol/L	
Triglyceride	1.7 mmol/L	
HDL	0.8 mmol/L	
LDL‐Calc	2.2 mmol/L	
Glucose	7.7 mmol/L	3.3–5.5

**FIGURE 2:**
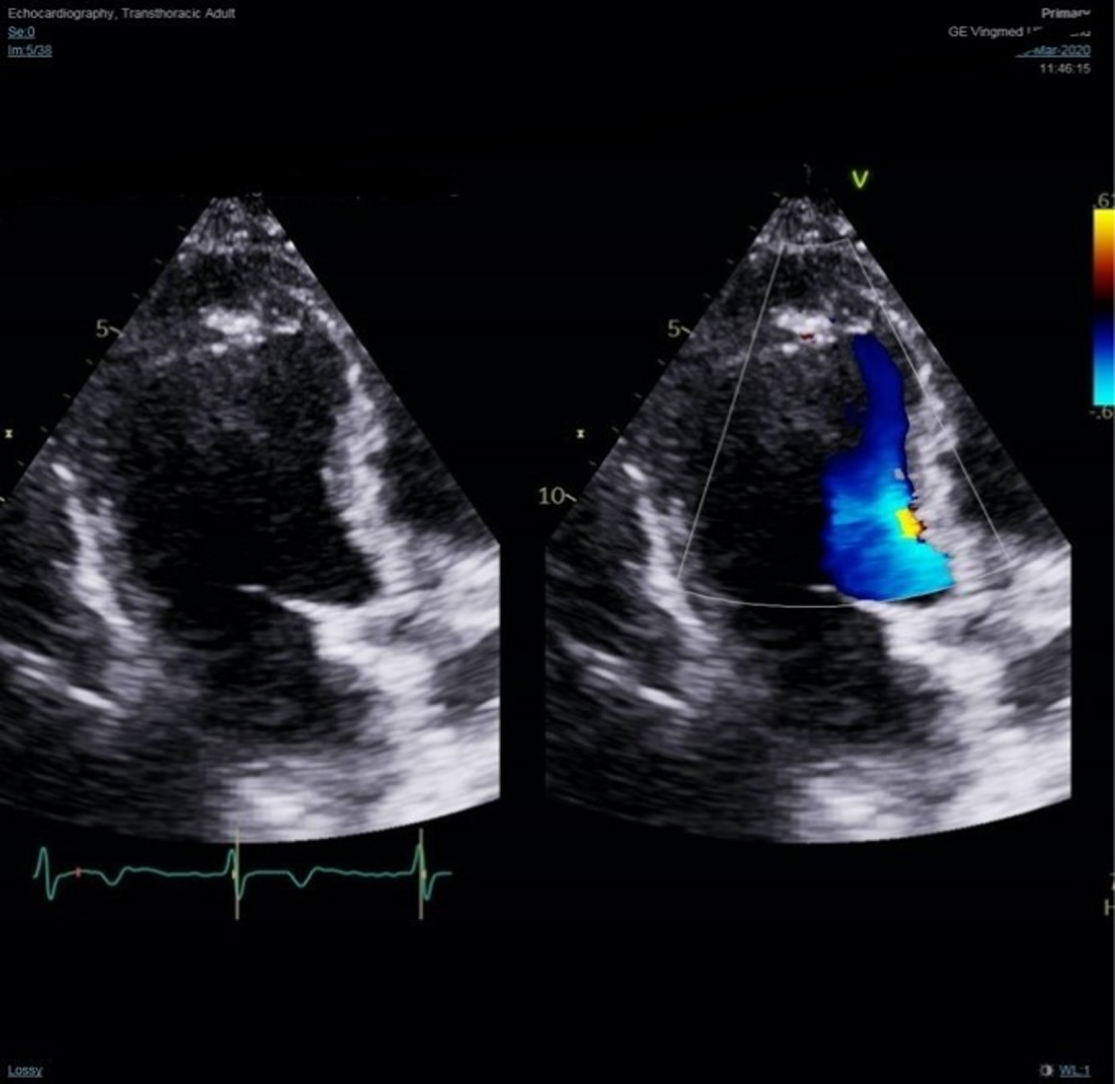
Echocardiography showed apical mass with calcification

A 2D transthoracic echocardiography was done 4 days later during the outpatient cardiology clinic follow‐up visit (Figure 2), which revealed; mildly reduced left ventricular (LV) systolic function with ejection fraction of 46%, asymmetric LV hypertrophy affecting the apical segments, which was aneurysmal. In addition, the apical cap contained a calcific material (calcified aneurysm vs. calcified thrombus), and Grade 1 diastolic dysfunction was noted. Further evaluation with CT coronary angiogram showed non‐significant left anterior descending artery lesion with concentric LV myocardial hypertrophy affecting the LV apex with subendocardial apical calcifications.

**FIGURE 3:**
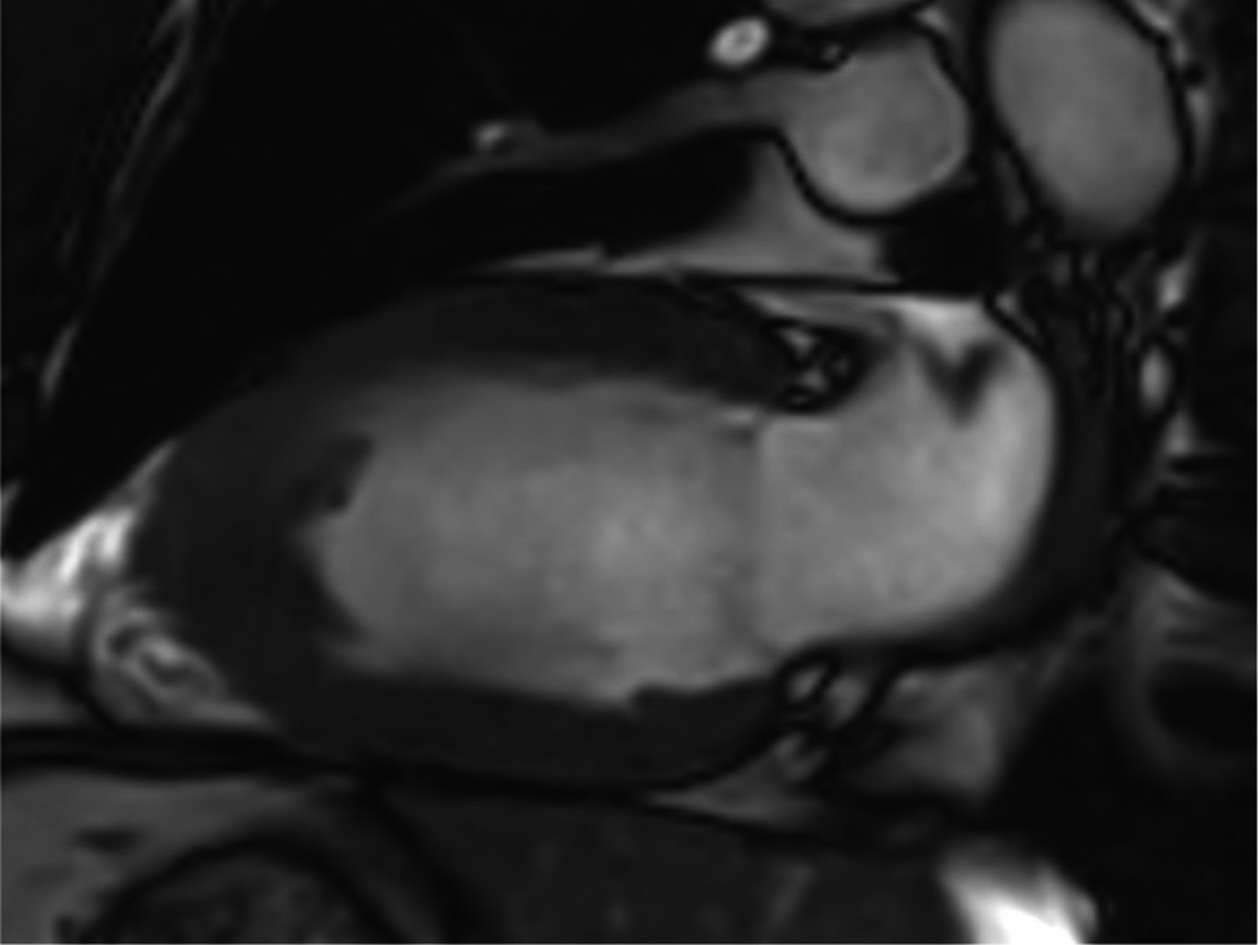
Two‐chamber view showing apical left ventricular thickening

**FIGURE 4:**
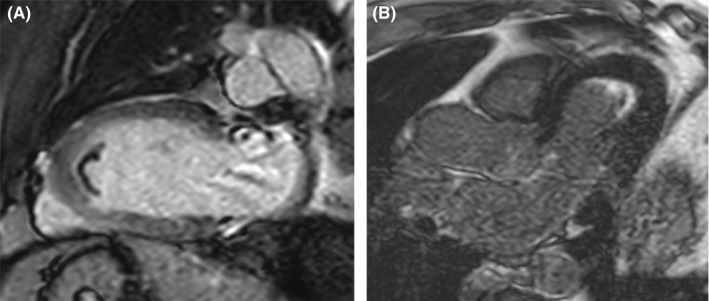
(A) Two‐chamber view showing apical filling of the left ventricle by thrombus exhibiting lower signal than adjacent myocardium at 2 min post‐gadolinium injection. (B) delayed sequences acquired in three‐chamber view 10 min after gadolinium injection showing subendocardial enhancement seen close to the thrombus

Cardiac MRI (CMR) images were performed later. Images obtained on a 1.5‐tesla scanner (Philips Ingenia) revealed thickening of the akinetic LV apical segments (Figure 3), which demonstrated non‐ischemic intense LV apical subendocardial late gadolinium enhancement (Figure 4). An apical LV thrombus was also noted. CMR concluded that the findings were consistent with EMF.

At this point, a diagnosis of EMF was made, and the patient was started on therapeutic dosing of anticoagulation in addition to Valsartan and Bisoprolol. Repeat blood works showed C‐reactive protein level 4.9 mg/L, and ANCA and Anti‐Schistosoma‐Ab were negative. Peripheral smear revealed normochromic, normocytic RBCs with few teardrop cells and mild rouleaux formation seen with mild absolute eosinophilia and occasional reactive lymphocytes. The patient continued to have non‐exertional, non‐radiating, on and off left‐sided chest pain. Hence an adenosine stress CMR perfusion study was performed. This CMR performed almost 6 weeks after the initial CMR showed no stress‐induced perfusion defects. Still, there was regression of the size of LV apical thrombus with no other apparent interval significant changes.

## 3 DISCUSSION

Endomyocardial fibrosis is a rare and newly emerging entity; it was first described in 1947 in Uganda. It is more commonly found in the developing world, such as in Africa, Asia, and South America.^1^ It is characterized mainly by fibrosis of the apical endocardium, mainly in the right, left, or both ventricles. The clinical manifestations usually arise from the symptoms of heart failure secondary to restrictive LV filling.^2^ The symptoms depend on the extent of involvement of the ventricles. The initial phase of the disease is acute carditis, characterized by febrile illness, and in some cases, it progresses to cardiogenic shock. But the vast majority present during the chronic phase with symptoms of heart failure, arrhythmia, and thromboembolism.^1^, ^4^

Generally, the main pathological event is unknown. However, there are multiple theories about its development, and further studies are needed to unfold its etiologies further and help us understand it better. Some of the ideas that have been postulated are (1) eosinophilia‐related process, which can lead to endocardial damage and fibrosis. (2) infection with prevalent organisms; however, no specific organism has been linked to its development. (3) autoimmune phenomenon has also been considered as some studies have found anti‐myosin antibodies, however, finding these antibodies is not specific, and lastly, (4) genetic components have been suggested. Still, none were completely linked to it.^5^

Diagnosis is usually challenging, as little is known about the true nature of the disease. However, there are several supporting features that we can depend on; ECG is normal in most cases; however, in advanced cases, it can show non‐specific ST‐T wave abnormalities, variable degrees of conduction block, or arrhythmias.^6^ Complete blood count can show eosinophilia which can further support the diagnosis, but it is usually absent.^7^ The exact role of eosinophilia in pathogenesis is poorly understood. Moreover, it is absence dictates that other factors might play a role. Echocardiography is the modality of choice while evaluating the disease; it can show apical obliteration, endocardial surface thrombi, or AV valve abnormalities.^8^ Another imaging modality currently gaining popularity is Cardiac MRI with gadolinium contrast enhancement which can demonstrate enhancement that further supports the diagnosis.^9^ Myocardial biopsy has been used before. However, a biopsy is invasive and has a high false‐negative rate. CMR can be utilized to diagnose this condition effectively. It offers the advantage of being non‐invasive as compared to myocardial biopsy.^10^

Treatment is often challenging, as there is little evidence on how to treat it. Standard medical therapy is to be used, such as diuretics for decongestion, ACEi⁄ARBs, and beta‐blockers, which can provide symptomatic relief. Still, the disease usually progresses in a short period. Anticoagulation is indicated for patients with proven endocardial thrombus.^11^ Surgery is one of the modalities that has improved survival in patients with advanced disease; the currently used approach includes endocardectomy combined with valvular replacement if the valve is damaged. However, it conveys a high mortality rate approaching 20%; recurrence after surgery has also been described. In severe advanced cases resistant to treatment, the last resort is heart transplantation.^12^ Prognosis is usually poor due to the progressive nature of the disease and the increased risk for complications such as arrhythmias and sudden cardiac deaths.^13^

## CONFLICT OF INTEREST

The authors have no conflict of interest to declare.

## AUTHOR CONTRIBUTIONS

Dr Wafer Dabdoob: Clinical care. Dr Ahmad Matarneh and Dr Yousif Alhailan: Clinical care, literature review, and manuscript write up. Dr Sabir Abdul Karim and Dr Maryam Al Kuwari: Imaging.

## ETHICAL APPROVAL

The case report was approved by Hamad medical corporation, MRC number MRC‐04–21–242.

## CONSENT

The authors have collected a written consent from the patient.

## Data Availability

The data that support the findings of this study are available from the corresponding author upon reasonable request.
